# Effects of Yoga Intervention on Functional Movement Patterns and Mindfulness in Collegiate Athletes: A Quasi-Experimental Study

**DOI:** 10.3390/ijerph192214930

**Published:** 2022-11-13

**Authors:** Dan Xu, Hua Wu, Hui Ruan, Cunzhu Yuan, Junke Gao, Meng Guo

**Affiliations:** 1 Faculty of Physical Education, Hainan Normal University, Haikou 571158, China; 2 Hainan Provincial Sports Academy, Haikou 571158, China

**Keywords:** yoga asanas, functional movement screen, Mindful Attention Awareness Scale, student athlete

## Abstract

High-quality movement patterns and high levels of mindfulness are thought to be beneficial in preventing sports injuries. Yoga is recommended in the field of athlete rehabilitation. This study investigated the effects of yoga intervention on functional movement patterns and mindfulness in collegiate athletes. It is a quasi-experimental study with a pre/post-test control design. The participants were divided into a yoga group and a control group. A Functional Movement Screen and the Mindful Attention Awareness Scale were used to assess participants’ basic movement patterns and mindfulness before and after 12 weeks of yoga intervention (two classes per week, 90 min per class). The results show that the yoga group’s FMS scores improved more compared to the control group [F(1,78) = 29.08, *p* < 0.001, ŋp^2^ = 0.27], and that the scores for the deep squat (ŋp^2^ = 0.4), shoulder mobility (ŋp^2^ = 0.17), and trunk stability pushup (ŋp^2^ = 0.36) improved substantially. The dysfunctional score ratio for deep squats (χ^2^ = 18.57, *p* < 0.001), shoulder mobility (χ^2^ = 26.90, *p* < 0.001), trunk stability pushup (χ^2^ = 17.07, *p* < 0.001), and rotatory stability (χ^2^= 38.29, *p* <0.001) decreased significantly compared with the control group, but there was no significant improvement in asymmetric movement patterns (χ^2^ = 0.75, *p* = 0.39). The mindfulness scores in the yoga group significantly exceeded those of the control group [F(1,78) = 13.56, *p* < 0.001, ŋp^2^ = 0.15]. These results suggest that yoga intervention can improve functional movement patterns and mindfulness levels, but further evidence is needed to determine whether yoga could positively influence sports injuries.

## 1. Introduction

According to the Sports Injury Monitoring system [1,2,3], student athletes have a high incidence of sports injuries. The occurrence of repeated and serious injury not only affects their development and causes a heavy physical and mental burden, but may also lead to the premature end of their sports career [4,5]. Therefore, the identification of sports injuries, their risk factors, and targeted interventions are important strategies for prevention [6].

A sports injury is a complex risk network system composed of multiple factors [7]. For student athletes, biomechanical changes [8,9], neurocognitive performance [10], physical performance, and movement pattern quality [11,12,13] have been identified as potential injury risk factors. The Functional Movement Screen (FMS) is a widely used tool to assess the quality of movement patterns. By assessing the flexibility, stability, and coordination of the body, it can analyze compensation, imbalance, and asymmetry in the subject’s movement [10]. Although the validity of FMS composite scores and cutoff scores for injury prediction remains controversial [11,12], movement asymmetry or poor-quality movement patterns have proven useful in estimating injury risk [13,14]. However, individual psychosocial factors such as stress, anxiety, and high levels of life event stress [15,16] could increase injury risk, but have been less targeted in sports injury research [6]. The National Athletic Trainers’ Association published a consensus on common psychological issues to monitor in collegiate athletes [17]. The prevalence of depression among these athletes is reported to be as common as in the general college population; in addition, they face sport-specific stressors [18,19]. Ivarsson et al. [15] suggested that stress responses and a history of stressors had the strongest correlation with injury rate among stress and sports injury models, and that psychological interventions should be considered when designing injury prevention programs. Ericksen et al. presented evidence in favor of athletic trainers/therapists incorporating psychological intervention strategies into injury prevention [20]. In contrast to traditional psychotherapy, which emphasizes the control and avoidance of negative emotions, emerging mindfulness therapy promotes tolerance, acceptance, and awareness of the present moment [21]. A recent review showed that mindfulness-based interventions have proven remarkably effective among student-athletes for increasing mindfulness, managing negative emotions and perceived stress, and improving overall wellbeing [22].

Yoga is a physical practice of mindfulness [21]. Yoga is an ancient science of mind and body movement that originated in India. It is a group of body and mind exercises that combines asanas, breathing, and meditation [23]. There is growing evidence to support the idea that yoga asanas improve health in different populations via downregulation of the sympathetic nervous system and hypothalamic–pituitary–adrenal system [24]. Ross et al. concluded that yoga may be as effective as or more effective than exercise in improving multiple factors related to both healthy and diseased populations’ health, including reducing stress and fatigue [25]. Yoga interventions are comparable to and/or superior to exercise in most outcome measures, while the emphasis on breathing, concentration, and the importance of maintaining posture distinguishes yoga from exercise [26]. Several studies have established that incorporating yoga interventions into daily training and exercise sessions of young athletes can enhance muscular functioning [27], and improve performance [28,29], physical fitness [30,31], and posture [32]. However, there are conflicting views about the effects of yoga. Owing to a lack of high-quality evidence, Breedvelt et al.’s meta-analysis suggested that the effectiveness of meditation, yoga, and mindfulness on depression, anxiety, and stress in college students should be interpreted with caution [33]. In Ravi’s [34] recent review on the application of yoga intervention in the rehabilitation and prevention of injury in competitive sports, which included only four studies, he suggested that it was possible to prevent non-contact injuries by including yoga programs in exercise training and that more research was needed. Consequently, this study evaluated the effects of yoga on movement patterns and mindfulness among collegiate athletes. Accordingly, we hypothesized that participants in the yoga group would achieve significantly higher FMS scores and substantially improved asymmetries and dysfunctional scores, and that their Mindful Attention Awareness Scale (MAAS) scores would remarkably increase after 12 weeks of yoga intervention.

## 2. Materials and Methods

### 2.1. Participants

Following the study by Eun Ju Lim and Jeong Eon Park [35] on sample size, we adopted our formula [36]. Assuming a detection level α = 0.05, the estimated loss to follow-up rate was 5%, which resulted in at least 39 participants in each group. As yoga classes were scheduled for sophomore year, this quasi-experimental study selected sophomore year students as the yoga group and freshman students as the control group. Recruitment was conducted in two sports training professional classes (freshmen class and sophomore class) in the Hainan Provincial Sports Academy in September 2021. The inclusion criteria were: (1) good health; (2) willingness to accept yoga instruction; and (3) lack of yoga experience. The exclusion criteria were: (1) yoga contraindications; (2) unwillingness to participate; (3) regular practice in special training courses or traditional Chinese health exercises such as Qigong and Tai chi; and (4) no musculoskeletal injuries at the time of testing.

A total of 80 participants took part in the experiment, with 39 in the yoga group (YOG group: 33 boys and 6 girls) and 41 (35 boys and 6 girls) in the control group (CON group). All the participants were college students majoring in sports training in the same training and learning environment. The YOG group participated in yoga training in addition to daily school life for 12 weeks. The CON group received no interventions to daily school life. The participant characteristics of each group are shown in Table 1 and Table 2. All the participants were similar in age, height, and weight, and there was a similar male-to-female ratio in each group. The study complied with the Declaration of Helsinki and was approved by the Ethics Committee of Hainan Provincial Sports Academy (GT-QM-01). All the participants signed written informed consent after understanding the trial procedures, risks, and benefits before participation.

### 2.2. Study Design and Procedures

#### 2.2.1. Procedure

**Figure 1:**
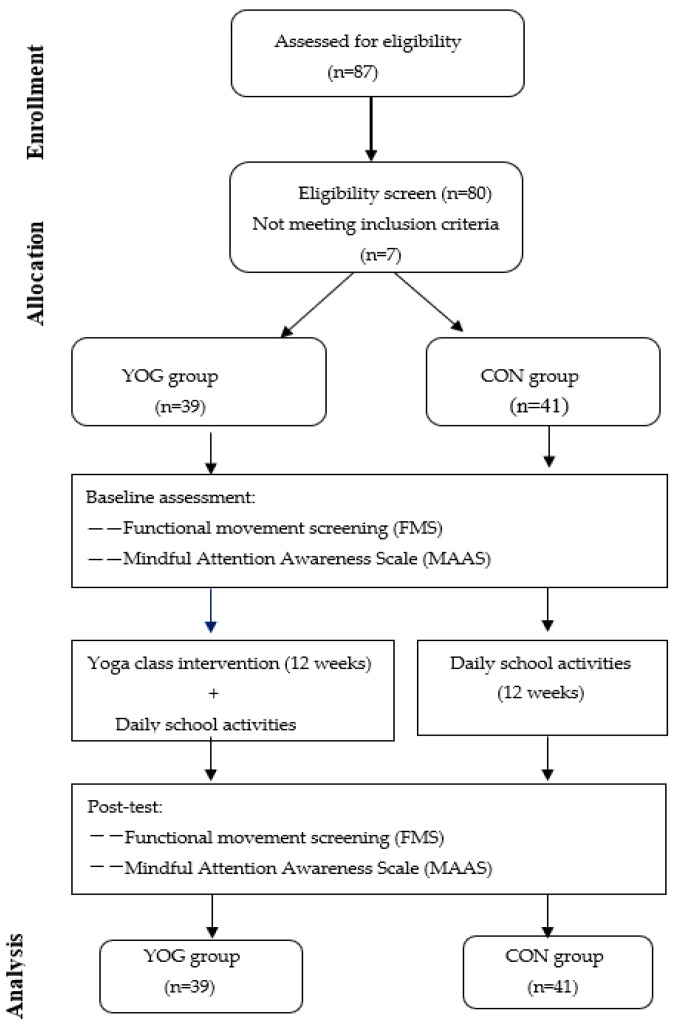
Study procedure. YOG group: yoga group; CON group: control group.

Before the intervention, the FMS and MAAS scores of the two groups were tested. After 12 weeks of yoga intervention (two classes per week, 90 min per class), the FMS and MAAS were retested per the pre/post-test control design (Figure 1). Data collection was conducted from September 2021 to January 2022.

#### 2.2.2. Intervention

The yoga intervention in this study was conducted by a professional yoga instructor in the yoga classroom on the third floor of Hainan Provincial Sports Academy twice a week, for 90 min each of yoga practice. The course content was divided into dynamic asanas and meditation static exercises. The dynamic exercises comprised a simple beginner’s version of the Ashtanga yoga primary sequence, which focuses on the six joints of the body (ankle, knee, hip, wrist, and elbow) and three-dimensional planes (horizontal, coronal, and sagittal planes). Each yoga class comprised meditation, yoga asanas, and relaxation techniques. The sessions were preceded by 10 min of stationary sitting meditation, followed by 70 min of yoga asanas, and 10 min of rest. Static meditation comprises stretching training of meditation and Yin yoga, including 30 min of sitting meditation and 50 min of shoulder and neck, spine, hip opening, and lower limb joint flexibility exercises, respectively (Table 3). During yoga meditation, college students were required to focus their attention and concentrate intently and continuously on the momentary sensations in the body, breath, consciousness, and a relaxed bodily state.

#### 2.2.3. Measurements

(1) FMS

The FMS is a special detection and evaluation method for basic movement patterns. This system aims to detect basic movements, motor control in movement patterns, and basic movement ability of people performing simple movements. The screening test consists of seven movements divided into two categories: the deep squat and trunk stability pushup are straight-pattern symmetrical tests; and the hurdle step, in-line lunge, active straight-leg raise, shoulder mobility, and rotary stability are split patterns [10]. The FMS test kit [37] was used by a qualitied rater employing standardized procedures, instructions, and scoring processes. Each movement is ranked among four scores according to the action standard (0, 1, 2, or 3), and the split patterns have two scores (left or right). The lowest score of each split pattern was recorded if the left and right scores were inconsistent; 21 points is the full composite score. Bonazza et al. [38] found that FMS showed excellent interrater and intrarater reliability.

(2) MAAS

The MAAS is a single-dimension self-report scale composed of 15 reverse items [39]. It is also one of the most used mindfulness measurement tools. Each item is scored on a 6-point Likert scale with frequency levels of 1 (almost always), 2 (very often), 3 (somewhat frequently), 4 (somewhat infrequently), 5 (very infrequently), and 6 (almost never), and the average score ranges from 15 to 90. The higher the average score, the higher the level of mindfulness. The internal consistency reliability of the original scale is between 0.72 and 0.81, while the reliability and validity of the Chinese version were tested by Chen et al. [40] on Chinese college students; the internal consistency reliability α coefficient was 0.89, and the internal test–retest reliability R coefficient was 0.87.

### 2.3. Statistical Analysis

The data were analyzed using SPSS 26.0 (IBM, Armonk, NJ, USA). The Kolmogorov–Smirnov test was used as the normal distribution test, and the various evaluation indexes were expressed as the mean standard deviation (M + SD) or frequency (ratio). The 2 (YOG and CON) × 2 (baseline and after 12 weeks) mixed-model ANOVA assessed the effect of the intervention on the FMS test (composite score, scores subset items) and MAAS results, and accessed between-group interaction effects (2 × 2 interaction effects). Simple main effects of time were analyzed to show the mean change in the FMS score after 12 weeks for each group, and the changes over 12 weeks were reported as a percentage (% Δ). The partial eta square (ŋp^2^) was reported as measure of the effect size for interaction effect and small (0.01), medium (0.06), and large (0.14) were defined. The ratio of split patterns (left-right symmetries or not) and individual item score classification (1 point or less, over 2 points) was analyzed using the chi-square test. The significance threshold was set at 0.05.

## 3. Results

### 3.1. FMS Scores of the Two Groups at Pre- and Post-Test

The results indicated that the yoga group’s FMS scores had significantly improved (% Δ = 30.08, *p* < 0.01) after 12 weeks of intervention and far exceeded the increase in the control group (% Δ = 11.27, *p* < 0.01). Further, the interaction effect of time and intervention was significant (F(1,78) = 29.08, *p* < 0.001, ŋp^2^ = 0.27). A comparison of the individual items in FMS scoring are presented in Table 4.

### 3.2. Asymmetrical and Dysfunctional Scores between the Two Groups at Pre- and Post-Test

Considering the importance of reporting the prevalence of asymmetrical and dysfunctional scores among adolescents [41], we analyzed the ratio of asymmetric and individual item scores of 1 point or less. The result showed that two groups had high asymmetric prevalence in the five separate items. The higher rates of asymmetry occurred in the in-line lunge (87.2%), rotatory stability (84.6%), and hurdle step (76.9%), and there were no significant differences at baseline and post-test (*p* > 0.05). Further, asymmetries appeared in at least one bilateral test in over 70% of the collegiate athletes at baseline in both groups (χ^2^ = 0.44, *p* = 0.51). The ratio dropped to 58% in the yoga group and 68% in the control group after the intervention, but there was still no significant difference (χ^2^ = 0.75, *p* = 0.39) (Table 5).

The results indicated that the ratio of dysfunctional scores was greater on rotatory stability (76.9%) and shoulder mobility (51%) at baseline in YOG and then decreased significantly in the deep squats (χ^2^ = 18.57, *p* < 0.001), shoulder mobility (χ^2^ = 26.90, *p* < 0.001), trunk stability pushup (χ^2^ = 17.07, *p* < 0.001), and rotatory stability (χ^2^ = 38.29, *p* < 0.001) after 12 weeks of yoga intervention (Table 6). Conversely, the control group showed a significant decline in the proportion of dysfunction scores in rotational stability and shoulder mobility only, as shown in Table 6.

### 3.3. The MAAS Scores at Pre- and Post-Test

Comparing the MAAS scores of the two groups after 12 weeks, the YOG scores improved more significantly than the CON (F(1,78) = 13.56, *p* < 0.001, ŋp^2^ = 0.15), and a significant interaction effect of time and intervention was indicated. The pre- and post-test changes within the group revealed that the YOG increased by 14.78% and demonstrated a remarkable statistical difference (*p* = 0.009 < 0.01) after 12 weeks of intervention. Although the baseline and post-test scores in the CON were higher than the YOG, the growth rate was small and statistically insignificant (*p* = 0.39) (Table 7).

## 4. Discussion

This study investigated the effects of yoga intervention on functional movement patterns and mindfulness in collegiate athletes. Our results show that compared to the control group, the yoga group significantly increased their FMS composite score, which was consistent with the findings of previous studies [35,42]. Meanwhile, we found that the 12-week yoga intervention significantly improved straight-pattern movements (deep squat and trunk stability pushup) and some split-pattern movements (all except hurdle step and in-line lunge). The yoga postures in this study were aimed at improving body flexibility, muscle endurance, joint flexibility, and core strength, which may explain the improved FMS total score. The FMS composite score to predict injury was 14 points, based on the first and often-cited article [43]. The yoga group’s FMS score increased from 13.0 to 17.0 (median), thus indicating that the injury risk of collegiate athletes was reduced after intervention.

However, in Pollen et al.’s critical review [13], the FMS composite score was positively correlated with the incidence of injury due to other risk factors that may have a greater impact on injury. A growing number of studies suggest that FMS composite scores are not related to injury [12] or should not be used in isolation to predict injuries [13]. Mokha et al. reported that athletes with asymmetrical scores or 1 point in a subset had twice the risk of musculoskeletal injury than others [14]; furthermore, the pain associated with FMS clearing tests may also be risk factors for injury [44]. Another study demonstrated a high prevalence of asymmetries in collegiate athletes, and over 70% of participants revealed asymmetries in at least one bilateral test; similar statistics have been reported in young male soccer players [45]. Although most of the yoga asanas are symmetrical in the direction of movement, such as forward bending and back bending, left and right bending, and left and right torsion in practice, our study revealed that the asymmetry rate in the yoga group did not significantly decrease after the intervention compared to the control group. This result may have been due to the collegiate athletes’ limb dominance and preference in asymmetric motion per their specific sports; thus, an obvious effect was not observed. However, the ratio of dysfunctional scores (≤1 point) on deep squats, shoulder mobility, trunk stability pushup, and rotatory stability improved significantly after the yoga intervention, thereby indicating that the intervention improved joint flexibility and core strength.

Mindfulness is defined as internal and external present-centered attention and awareness. It is also a simple way to reduce pain and transform negative emotions, which can effectively affect the mental health of athletes. Wu et al. suggested that mindfulness was positively associated with psychological skills and mental toughness related to sports performance [46]. This study found that the yoga group’s MAAS scores significantly increased following the intervention compared to the control group, showing a higher level of mindfulness and consistency with the expected results. Studies on sports teams have reported that after yoga intervention, athletes exhibited better mindfulness and greater goal-directed energy [21]. Arambulo et al. [47] proposed that when practitioners focused on the present moment during yoga meditation, the body would enter a deep state of relaxation to calm the parasympathetic nervous system, thereby reducing the respiratory rate, blood pressure, heart rate, oxygen consumption, body pressure, and the effect of brain waves. The course content of the yoga intervention used in this study comprised asanas and meditation. Thus, our results supported the hypothesis that yoga intervention could improve mindfulness.

### Limitations

This study had several limitations. Although a clear potential effect of yoga intervention on mindfulness and functional movement levels was identified, many issues need to be addressed. First, the participants partook in different sports, and the yoga classes used in this study were not targeted at specific or individual weaknesses; therefore, the effectiveness of the intervention may have been influenced. Second, the participants in the yoga group received mindfulness meditation content, but the control group received no intervention. The result of MAAS could be affected by this bias. Owing to a lack of statistics on the epidemiology of sports injuries, no stronger evidence can be drawn. Future studies should take these limitations into account and randomized controlled trials are necessary.

## 5. Conclusions

Yoga intervention has proven more efficient for FMS composite scores and in significantly reducing the rate of dysfunctional scores on deep squats, shoulder mobility, trunk stability pushup, and rotatory stability after the intervention, but the improvement was not significant for asymmetrical movement patterns. Moreover, yoga intervention has been shown to help increase levels of mindfulness in collegiate athletes. Future studies should design targeted yoga interventions for specific projects or individual weaknesses to further explore its effects and potential to prevent sports injuries. Another direction could be to explore the impact of yoga interventions on athletes at different levels.

## Figures and Tables

**Table 1:** Basic information of the participants (M ± SD).

Group	N	Age (Year)	Height (cm)	Weight (kg)
YOG	39	17.7 ± 0.8	171.2 ± 7.3	61.2 ± 10.8
CON	41	16.4 ± 0.5	171.0 ± 6.1	59.6 ± 9.40

**Table 2:** Sport-specific information sheet.

Group	Basketball	Football	Volleyball	Athletic	Tennis	Badminton	Sanda	Taekwondo	Total
YOG	6	4	6	8	2	4	3	6	39
CON	9	4	14	3	3	4	0	4	41

**Table 3:** Yoga intervention content.

Week	No.	Content
1st	①	Sun salutation A
	②	Mindfulness meditation for 30 min, neck-shoulder theme: Bowing pose, Eagle, Cow-faced pose, Reverse praying, Eye of the needle pose, Heart melting pose, Twisted root, Open wing, Corpse pose
2nd	③	Repeat ①, Surya Namaskara
	④	Mindfulness meditation for 30 min, Repeat ②
3rd	⑤	Repeat ③, Standing Postures: Standing forward bend, Triangle, Extended triangle, Side angle, Extended side angle, Corpse pose
	⑥	Mindfulness meditation for 30 min, Spinal theme: Crow, Cat, Seal, Sphinx, Tadpole, Heart melting pose, Corpse pose
4th	⑦	Repeat ⑤, Standing Postures: Standing straddled forward bend ABCD, Intense side stretch pose, Half-lotus bend, Chair pose, Warrior 1, Warrior 2, Corpse pose
	⑧	Mindfulness meditation for 30 min, Repeat ⑥
5th	⑨	Repeat ⑦, Sitting Postures: Seated mountain pose, Staff pose, Inclined plane pose, Intense stretch to the west pose ABC, Corpse pose
	⑩	Mindfulness meditation for 30 min, Hip opening theme 1: Wandering dragon, twisting dragon, Royal pigeon pose, Eye of a needle type, Frog, Cradle pose, Marichi pose, Supine twist, Royal pigeon pose, Bound angle, Corpse pose
6th	⑪	Repeat ⑨, Sitting Postures: Half hero forward bend, Three limbs face one foot pose, Fire log, Seated back stretching pose ABC, Corpse pose
	⑫	Mindfulness meditation 30 min, Repeat ⑩
7th	⑬	Repeat ⑪, Sitting Postures: Marichyasana ABCD, Boat, Corpse pose
	⑭	Mindfulness meditation for 30 min, Hip opening theme 2: Cow-faced pose, Dragon, Low lunge, Seated angle pose, Monkey hanumanasana split pose, Happy baby, Square, Bound angel pose, Corpse pose
8th	⑮	Repeat ⑬, Finishing Postures: Wheel pose, Corpse pose
	⑯	Mindfulness meditation for 30 min, Repeat ⑭
9th	⑰	Repeat ⑮, Finishing Postures: Shoulder stand, Plough pose, Fish pose, Spinal twist, Corpse pose
	⑱	Mindfulness meditation for 30 min, Lower limbs stretch theme: Low lunge, Halfmoon, Half hanumanasana split, Low arch twist and stretch quadriceps pose, Corpse pose
10th	⑲	Repeat ⑰, Finishing Postures: Hands bound head stand, Corpse pose
	⑳	Mindfulness meditation for 30 min, Repeat ⑱
11th	㉑	Repeat ⑲, Lotus pose, Corpse pose
	㉒	Mindfulness meditation for 30 min, Lower limbs stretch theme: Supine hero pose, Supine hand grab big toe pose, Triangle, Seated forward bends, Sitting glissade, Wide leg sitting forward bend, Pigeon king pose, One leg head to knee, Triangle proneness, Corpse pose
12th	㉓	Repeat ⑳, Complete sequence Repeat
	㉔	Mindfulness meditation 30 min, Ankle joint theme: Hero’s, Toe touch heroes sit, squat toe touch pose, Squat balance pose, Bound squat

Note: Flow dynamic stretch + supine body scan exercise in the week’s first class, and meditation + static stretch exercise in the week’s second class.

**Table 4:** FMS scores at pre-and post-test.

Outcome	Group	Pre-Test	Post-Test	% Δ	A Group-by-Time Interaction Effect		
		Mean ± SD	Mean ± SD		F(1,78)	*p*	Partial ŋ^2^
Deep Squat							
	YOG	1.67 ± 0.81	2.77 ± 0.43	65.87 **	52.33	<0.001	0.40
	CON	2.17 ± 0.59	2.12 ± 0.84	−2.30			
Hurdle Step							
	YOG	1.95 ± 0.39	2.03 ± 0.71	4.10	0.282	0.60	0.004
	CON	1.63 ± 0.54	1.63 ± 0.58	0			
In-line Lunge							
	YOG	2.69 ± 0.52	2.64 ± 0.54	−1.86	3.74	0.06	0.05
	CON	2.54 ± 0.55	2.20 ± 0.72	−13.39 **			
Shoulder Mobility							
	YOG	1.74 ± 0.85	2.64 ± 0.49	51.72 **	15.43	<0.001	0.17
	CON	1.83 ± 0.77	2.07 ± 0.82	13.11 *			
Active Straight-Leg Raise							
	YOG	2.08 ± 0.74	2.67 ± 0.62	28.37 **	0.85	0.36	0.01
	CON	1.66 ± 1.06	2.44 ± 1.01	47.99 **			
Trunk Stability Pushup							
	YOG	1.59 ± 0.68	2.31 ± 0.47	45.28 **	44.44	<0.001	0.36
	CON	1.95 ± 0.38	1.98 ± 0.35	1.53			
Rotatory Stability							
	YOG	1.23 ± 0.43	1.92 ± 0.27	56.10 **	0.15	0.70	0.002
	CON	1.05 ± 0.22	1.78 ± 0.42	69.52 **			
Total FMS							
	YOG	13.03 ± 2.41	16.95 ± 1.89	30.08 **	29.08	<0.001	0.27
	CON	12.78 ± 2.06	14.22 ± 2.31	11.27 **			

* *p* < 0.05, ** *p* < 0.01.

**Table 5:** FMS asymmetric movement patterns at pre- and post-test.

Identified Asymmetric Movement Pattern		Yes n%		No n%		χ^2^	*p*
Pre-test	YOG	8	20.5	31	79.5	0.44	0.51
	CON	11	26.8	30	73.2		
Post-test	YOG	16	41.0	23	59.0	0.75	0.39
	CON	13	31.7	28	68.3		

**Table 6:** The yoga group’s FMS dysfunctional scores ratio at pre- and post-test for the two groups.

	Yoga Group					Control Group			
Outcome	Time	≤1 Point [n (%)]	≥2 Point [n (%)]	χ^2^	*p*	≤1 Point [n (%)]	≥2 Point [n (%)]	χ^2^	*p*
Deep Squat									
	Pre-test	15 (38.5)	24 (61.5)	18.57	<0.001 **	4 (9.8)	37 (90.2)	0	1
	Post-test	0 (0)	39 (100)			4 (9.8)	37 (90.2)		
Hurdle Step									
	Pre-test	4 (10.3)	35 (89.7)	2.31	0.13	16 (39)	25 (61.0)	0.05	0.82
	Post-test	9 (23.1)	30 (76.9)			17 (41.5)	24 (58.5)		
In-line Lunge									
	Pre-test	1 (2.6)	38 (97.4)	0	1	1 (2.4)	40 (97.6)	4.99	0.03
	Post-test	1 (2.6)	38 (97.4)			7 (17.1)	34 (82.9)		
Shoulder Mobility									
	Pre-test	20 (51.3)	19 (48.7)	26.90	<0.001 **	16 (39.0)	25 (61.0)	6.21	0.01 **
	Post-test	0 (0)	39 (0)			6 (14.6)	35 (85.4)		
Active Straight-Leg Raise									
	Pre-test	7 (17.9)	32 (82.1)	1.84	0.18	12 (29.3)	29 (70.7)	1.71	0.19
	Post-test	3 (7.7)	36 (92.3)			7 (17.1)	34 (82.9)		
Trunk Stability Pushup									
	Pre-test	14 (35.9)	25 (64.1)	17.07	<0.001 **	4 (9.8)	37 (90.2)	0.16	0.69
	Post-test	0 (0)	39 (100)			3 (7.3)	38 (92.7)		
Rotatory Stability									
	Pre-test	30 (76.9)	9 (23.1)	38.29	<0.001 **	39 (95.1)	2 (4.9)	45.22	<0.001 **
	Post-test	3 (7.7)	36 (92.3)			9 (22.0)	32 (78.0)		

** *p* < 0.01.

**Table 7:** MAAS scores at pre- and post-test.

Outcome	Group	Pre-Test	Post-Test	% Δ	A Group-by-Time Interaction Effect		
MAAS		Mean ± SD	Mean ± SD		F(1,78)	*p*	Partial ŋ^2^
	YOG	53.62 ± 15.36	61.54 ± 12.31	14.78 **	13.56	<0.001	0.15
	CON	65.93 ± 14.53	67.80 ± 13.00	2.84			

**: *p* < 0.01.

## Data Availability

The data presented in this study are available in Supplementary Material.

## Supplementary Materials

The supporting information can be downloaded at: https://www.mdpi.com/article/10.3390/ijerph192214930/s1.
